# Paratesticular Adenomatoid Tumor Mimicking Testicular Malignancy: A Case Report and Literature Review

**DOI:** 10.7759/cureus.74935

**Published:** 2024-12-01

**Authors:** Abdulmalik G Abumohssin, Ghaida Daghistani, Abdelrazak Meliti, Reem Alfaraj, Islam Junaid, Abdulaziz Khiyami

**Affiliations:** 1 Radiology, King Faisal Specialist Hospital and Research Centre, Jeddah, SAU; 2 Radiology, King Abdullah Medical Complex, Jeddah, SAU; 3 Pathology, King Faisal Specialist Hospital and Research Centre, Jeddah, SAU; 4 Urology, King Faisal Specialist Hospital and Research Centre, Jeddah, SAU

**Keywords:** adenomatoid tumor, extra-testicular tumor, paratesticular lesion, paratesticular tumor, radical orchiectomy

## Abstract

Paratesticular adenomatoid tumors are benign and rare neoplasms, and the management of these lesions is challenging as many cases end up in the operation room due to the lack of specific clinical and radiological features to differentiate them from malignant lesions. We report a case of adenomatoid tumor of the tunica albuginea in a 48-year-old male along with a review of the literature for similar cases in the last 10 years.

## Introduction

Intra-scrotal tumors encompass a diverse group of neoplastic lesions, with paratesticular tumors representing a relatively rare subset, accounting for only about 5% of all cases [1]. Paratesticular tumors originate from structures adjacent to the testis, such as the epididymis, spermatic cord, tunica albuginea, and the surrounding mesothelial tissues. Among these, adenomatoid tumors are notable for being benign yet rare mesothelial-origin neoplasms, comprising approximately 30% of all paratesticular masses [2]. Despite their benign nature, adenomatoid tumors can present a significant diagnostic challenge due to their nonspecific clinical and radiological features. Potential anatomical locations for these tumors include the epididymis, which is the most common site, followed by the tunica albuginea, spermatic cord, and, in rare instances, the testicular parenchyma [3]. This variability in location can further complicate the clinical assessment and diagnosis.

From a radiological perspective, the identification of adenomatoid tumors can be problematic as there are no definitive imaging characteristics that distinguish them from other benign or malignant intra-scrotal masses. Ultrasound, the primary imaging modality for scrotal evaluation, often reveals a well-circumscribed, hypoechoic mass; however, these features are not unique to adenomatoid tumors and can overlap with those seen in malignant lesions such as sarcomas or metastatic deposits [4]. As a result, the diagnostic accuracy of ultrasound in identifying adenomatoid tumors remains limited, necessitating further investigation through advanced imaging or histopathological evaluation.

Clinically, the presentation of adenomatoid tumors is often subtle, with many patients being asymptomatic. When symptoms do arise, they typically include mild scrotal discomfort, swelling, or the presence of a palpable mass. These symptoms can mimic those of more common conditions such as epididymitis, hydrocele, or malignant tumors, potentially leading to misdiagnosis or unnecessary surgical intervention [5]. Given the rarity and atypical presentation of adenomatoid tumors, clinicians must maintain a high index of suspicion, especially when evaluating paratesticular masses with uncertain etiology.

The purpose of this study is to draw attention to the clinical and radiological features of paratesticular adenomatoid tumors. This entity remains under-recognized in both urological and radiological practice. Furthermore, this study aims to comprehensively review the existing literature, focusing on cases reported over the past 10 years. By analyzing keywords related to adenomatoid tumors, this review seeks to consolidate the current understanding of the epidemiology, clinical presentation, imaging findings, and management strategies to improve diagnostic accuracy and patient outcomes.

## Case presentation

A 48-year-old male, with no previous medical or surgical issues, was sent to the urology department due to a testicular mass he had noticed for the past two months. He reported no symptoms related to urination or general health and had not experienced any previous injuries. His family history was negative for testicular cancer.

**Figure 1:**
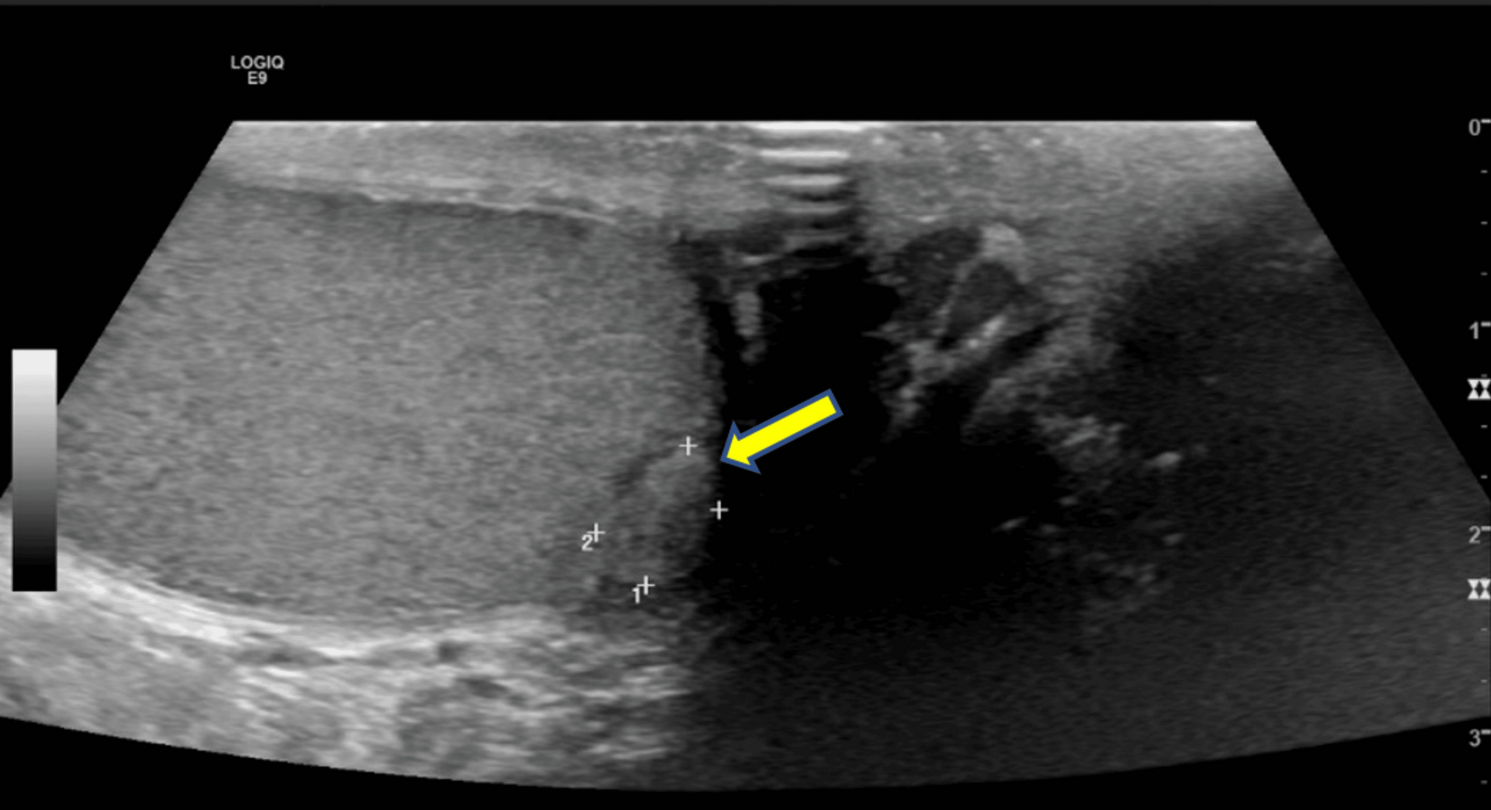
Scrotal ultrasound showing the left intratesticular peripheral hypoechoic lesion measuring 0.7 x 0.6 cm with no internal vascularity (yellow arrow).

Upon examination, his vital signs were normal. The physical exam showed a firm, non-tender mass in his left testicle, which was not associated with enlarged lymph nodes. Further investigation through a scrotal ultrasound showed a solid, hypoechoic rounded mass in the lower pole of the left testicle, measuring about 0.7 cm x 0.6 cm (Figure 1). No internal vascularity within the mass or hydrocele was observed. The right testicle appeared normal in the ultrasound.

**Figure 2:**
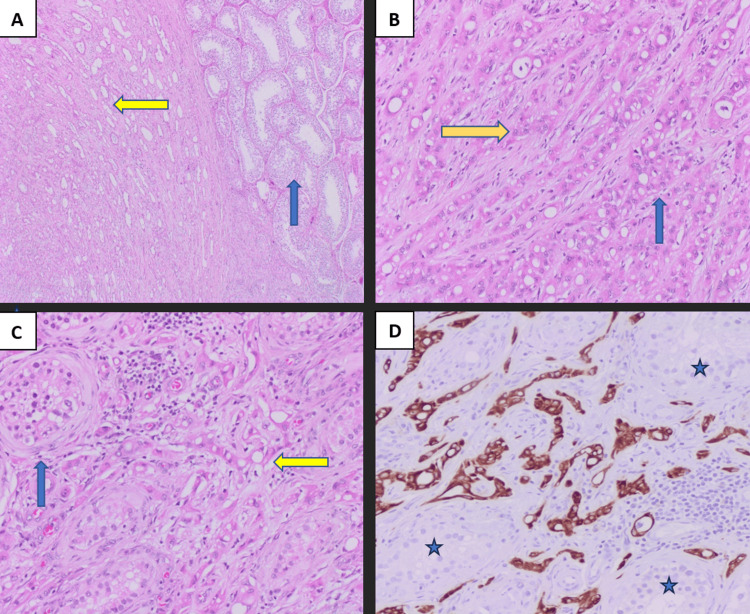
Hematoxylin and eosin-stained section and CAM 5.2 immunohistochemistry marker. (A) Hematoxylin and eosin-stained section (at 100X), showing normal seminiferous tubules (blue arrow) and cellular proliferation with a pseudoglandular pattern (yellow arrow). (B) Hematoxylin and eosin-stained section (at 200X), demonstrating neoplastic cells with eosinophilic cytoplasm and trabecular/corded (yellow arrow) and pseudoglandular growth patterns (blue arrow). (C) Hematoxylin and eosin-stained section (at 200X), demonstrating a focal infiltrative pattern of neoplastic cells (yellow arrow) between seminiferous tubules (blue arrow). (D) Cytokeratin (CAM 5.2) immunohistochemistry stain highlighting the neoplastic cells permeating between seminiferous tubules.

Further imaging included thoracic and abdominopelvic cross-sectional imaging, which was negative for lymphadenopathy and distal metastasis. Hormonal profiles, including luteinizing hormone (LH), follicle-stimulating hormone (FSH), testosterone, a-fetoprotein (AFP), and beta-chorionic gonadotropin, were all within normal limits. As the lesion was concerning for malignancy radiologically and clinically, the patient was counseled and booked for elective orchiectomy. The operation was successful, and the patient was discharged after 24 hours in stable condition. Grossly, the mass showed a well-circumscribed tan, white firm mass. The histopathology showed a focal infiltrative pattern of neoplastic cells between seminiferous tubules (Figure 2), while the immunohistochemistry markers (Table 1) showed positive calretinin, Wilms tumor marker (WT-1), and anticytokeratin monoclonal antibodies 1 and 3 (AE1/AE3).

**Table 1:** Immunohistochemistry marker findings. WT-1: Wilms tumor marker; CAM 5.2: Cell marque cytokeratin; AE1/AE3: Anticytokeratin monoclonal antibodies; 'D2-40: Monoclonal antibodies; CD31, CD34: Cluster of differentiation; SALL4: Spalt-like transcription factor 4

Stain	Result
Calretinin	Positive
WT-1	Positive
CAM 5.2	Positive
AE1/AE3	Positive
D2-40	Positive
CD31	Negative
CD34	Negative
SALL4	Negative

## Discussion

Structures within the scrotal area outside of the testis itself are referred to as extra- or paratesticular in location; tumors within this compartment are uncommon worldwide, accounting for less than 5%; the latest literature shows that this type of tumor originates from mesothelial cells and does not depend on hormones [6]. Adenomatoid tumors (AT) are the predominant type found in the paratesticular region, making up around 30% of all masses in this area; they were initially identified by Golden and Ash back in 1945; the most common site is the epididymis, followed by the tunica albuginea and least within the testicular parenchyma [7].

Ten case reports that were accessible and English-written were identified in the last 10 years. They were searched using PubMed, Google Scholar, ScienceDirect, and a manual review of references. The terms utilized were "extratesticular AT" OR "paratesticular AT" OR "AT ultrasound" OR "AT mimicking carcinoma" OR "paratesticular AT orchiectomy" (Table 2).

**Table 2:** Clinical, radiological, and histopathological characteristics of scrotal adenomatoid tumors in published cases within the last 10 years.

	Case 1	Case 2	Case 3	Case 4	Case 5	Case 6
Author	Guo et al.^7^	Patoulias et al.^12^	Pichler et al.^4^	Nham^5^	Abdullah^8^	Efared^10^
Year of publication	2015	2016	2018	2020	2020	2022
Age (years)	12	16	51	39	60	47
Side	Left	Right	Left	Left	Right	Right
Location	Tunica Albuginea	Epidydimal	Tunica Albuginea	Epidydimal	Epidydimal	Epidydimal
Initial symptoms	Swelling, pain	Swelling	Not provided	Swelling, Pain	Swelling	Swelling, Pain
Imaging workup	US	US	US, CT	US, CT	US	US, CT
Size in mm	0.8 x 1.0	1.3 x 1.1	0.9 x 0.9	13	2 X 1.9	10 x 10
Echogenicity	Hypoechoic	Hyperechoic	Hyperechoic	Hypoechoic	Hypoechoic	Hyperechoic
Tumor markers	Not provided	Negative	Negative	Negative	Negative	Negative
Intraoperative frozen section	Done	Not done	Done	Not done	Not done	Not done
Tissue invasion	-	-	+	-	-	-
Immunohisto-chemistry	Calretinin, Cytokeratin Vimentin	HMB1, Calretinin	Not provided	Mesothelial markers	Calretinin, HMB-1	Not performed
Management	Tumor resection	En block excision	Organ-sparing surgery	Radical orchiectomy	Local resection	Organ sparing surgery
	Case 7	Case 8	Case 9	Case 10	Case 11	
Author	Bhattu et al^9^	Dighe et al^1^	Corvino et al^11^	Farah et al^15^	Abumohssin	
Year of publication	2022	2022	2023	2023	2024	
Age (years)	40	40	60	57	48	
Side	Left	Left	Left	Right	Left	
Location	Testicular	Epidydimal	Epidydimal	Epidydimal	Tunica Albuginea	
Initial symptoms	Swelling	Swelling	Swelling	Swelling	Swelling	
Imaging workup	US, CT	US	US	US	US, CT	
Size in mm	Not provided	30 x 30	4.5 x 4.1	12 x 10	0.7 X 0.6	
Echogenicity	Hypoechoic	Isoechoic	Hyperechoic	Hyperechoic	Hypoechoic	
Tumor markers	Negative	Not sought	Negative	Negative	Negative	
Intraoperative f rozen section	Not done	Done	Not done	Not done	Not done	
Tissue invasion	-	-	-	-	+	
Immunohisto-chemistry	D2-40, WT-1, Calretinin	Calretinin	WT-1, Calretinin, Cytokeratin, AE1/AE3, EMA	CK 5/6, WT-1	Calretinin, Cytokeratin, WT-1	
Management	Radical orchiectomy	En bloc excision	Radical orchiectomy	Epididym- ectomy	Radical orchiectomy	

The youngest reported patient was a 12-year-old boy by author Guo et al. The oldest were two male patients aged 60 by author Xing and Bhattu et al; in this case, the patient was 48, considered within the common age groups [7-9]. The most common clinical presentation was painless swelling, similar to the current case. However, three reported cases experienced pain [5,7,10].

Routine workup includes laboratory work, scrotal ultrasound, and cross-sectional imaging; all reported cases had normal tumor markers and no evidence of intra-abdominal metastasis; as for ultrasound findings, variable measurements, and echo patterns were documented; however, none were too specific to differentiate AT from other benign or malignant lesions. No site predilection was found, given that a sonogram is still the gold standard exam for all scrotal masses [1,8].

Operative management is usually taken for all intra-scrotal lesions, as with this case, the surgeon’s main concern was testicular carcinoma, and thus, radical orchiectomy was done; previous authors such as Guo et al. [7] took the decision for the frozen section after visually inspecting the tumor arising from the tunica albuginea, Dighe et al. [1] after detecting the lesion in the head of the epididymis, and the only report by Pichler et al. [4] stating the use of contrast ultrasound that showed enhancement of the lesion.

The final definitive diagnosis requires histopathology and immunohistochemistry; as in this case, markers were positive for calretinin, WT-1, CAM5.2, AE1/AE3, and D2-40; the most common and specific reported markers in the literature were calretinin and WT-1 (2), and other reported markers include HMB-1 and cytokeratin [7,8,11,12]. Despite the benign nature of AT, pathology samples showed an element of tissue infiltration into the testicular parenchyma, and this has been reported in one case where the tumor originated from the tunica ambiguine and infiltrated into the testicular parenchyma [12]. The differential diagnosis for paratesticular AT includes malignant mesothelioma, metastatic adenocarcinoma, and testicular malignancy [13].

MRI with contrast has been suggested by Goel et al. and Farah et al. to localize the lesion better and identify the pattern of enhancement, which improves the detection of possible benign lesions [14,15].

## Conclusions

Paratesticular adenomatoid tumors are a rare entity with variable clinical presentations and imaging patterns. The initial workup includes a scrotal ultrasound, CT of the abdomen and pelvis, and tumor markers. The surgical approach is widely accepted, and histopathological features are the mainstay of diagnosis. Current literature does not show a specific identification of this lesion, so avoiding unnecessary radical orchiectomies remains challenging for future cases. Future studies are encouraged to investigate the variable patterns of this lesion.
